# Analysis and comparison of the trends in burden of malignant cutaneous melanoma in East Asian countries and regions and worldwide from 1991 to 2021

**DOI:** 10.3389/fpubh.2025.1487177

**Published:** 2025-04-30

**Authors:** Jiaxiang Xu, Xiaoyu Wang, Wenhui Liu, Xuanjun Liu, Guangshuai Li

**Affiliations:** The First Affiliated Hospital of Zhengzhou University, Zhengzhou, China

**Keywords:** malignant melanoma, trend, incidence, mortality, prevalence, disability-adjusted life years

## Abstract

**Objectives:**

This study aims to comprehensively analyze temporal trends in the burden of malignant melanoma (MM) in East Asia, focusing on incidence, prevalence, mortality, and disability-adjusted life years (DALYs) from 1991 to 2021. It further seeks to compare these trends with the global burden of disease.

**Study design:**

The study utilized data from the Global Burden of Disease (GBD) database to examine the disease burden of MM across East Asian countries and regions, as well as globally, over a 30-year period (1991–2021).

**Methods:**

We assessed changes in the incidence, prevalence, mortality, and DALYs associated with MM in East Asia and globally using GBD database open-source data. To capture the underlying trends in the disease burden, we applied the Joinpoint regression model to calculate the average annual percentage change (AAPC) and corresponding 95% confidence intervals (95% CI). A detailed comparative analysis was conducted to explore differences in the burden of MM across East Asian regions and compared with global trends, with particular emphasis on age, sex, and temporal changes.

**Results:**

The greatest increase in MM incidence in East Asia was observed in Korea, where the age-standardized incidence rate (ASIR) rose from 0.603 cases per 100,000 population (95% CI: 0.389–0.789) in 1991 to 1.896 cases per 100,000 (95% CI: 0.78–2.499) in 2021. Regarding prevalence, China exhibited the most significant increase in East Asia, with the age-standardized prevalence rate (ASPR) increasing from 0.699 (95% CI: 0.451–0.864) per 100,000 in 1991 to 4.157 (95% CI: 2.195–5.633) per 100,000 in 2021. The highest increases in MM mortality and DALYs were noted in Taiwan Province of China, where the age-standardized mortality rate (ASMR) increased from 0.36 (95% CI: 0.339–0.382) per 100,000 in 1991 to 0.414 (95% CI: 0.414) per 100,000 in 2021. Similarly, the age-standardized DALY rate (ASDR) in Taiwan rose from 10.375 (95% CI: 9.781–11.049) per 100,000 in 1991 to 11.647 (95% CI: 10.558–12.478) per 100,000 in 2021. Age and gender exhibited distinct patterns of influence on the MM burden: while ASIR generally increased with age, ASPR initially increased and later plateaued. Both ASMR and ASDR demonstrated a positive correlation with age. Additionally, male populations consistently exhibited higher morbidity and mortality rates than females.

**Conclusion:**

Over the period from 1991 to 2021, there were significant variations in the incidence, prevalence, mortality, and DALY rates of MM across East Asian countries and regions, including China, Japan, South Korea, North Korea, and Taiwan. These disparities underscore the need for region-specific, proactive prevention strategies and targeted public health interventions to mitigate the growing burden of malignant melanoma in the region.

## Introduction

Skin cancers are known for their aggressive nature, with malignant melanoma (MM) being the most lethal among them (1). Malignant melanoma originates from melanocytes, specialized skin cells responsible for producing pigment. It is characterized by highly malignant tumors that result from the abnormal proliferation of melanocytes, driven by a combination of genetic and environmental factors. While less common than basal cell carcinoma (BCC) and squamous cell carcinoma (SCC) in terms of morbidity, melanoma is more dangerous due to its propensity for rapid metastasis to other organs and systems, particularly if not diagnosed and treated in its early stages (2).

Unlike many other cancers, melanoma predominantly affects adolescents and middle-aged adults. Its incidence tends to rise between the ages of 25 and 50, after which it declines, particularly in women. However, in individuals over the age of 55, melanoma incidence is higher in men compared to women. The global incidence of malignant cutaneous melanoma continues to rise, with an annual increase of approximately 2.6%. In 2020, new cases of MM accounted for 1.68% of all newly diagnosed cancers (approximately 325,000 cases), while deaths from MM represented 0.57% of all cancer-related fatalities that year (3). When diagnosed and treated early, the five-year survival rate for patients with melanoma is as high as 99%. In stark contrast, individuals diagnosed with stage IV melanoma often face a grim prognosis, with survival rarely extending beyond one year (4). As the global population ages, the burden of melanoma has grown, leading to an increasing number of MM-related cases worldwide. This trend has made melanoma a significant public health concern due to its high mortality, recurrence, and metastasis rates, which result in substantial health and economic burdens for individuals, families, and society at large.

The Global Burden of Disease (GBD) database, compiled by the Institute for Health Metrics and Evaluation (IHME) at the University of Washington, USA, provides comprehensive, global disease-related data. The most recent update to the database is from 2021, making it the most extensive and reliable resource for tracking the global burden of disease (5). Previous studies on malignant melanoma (MM) using the GBD database have shown that, like most cancers, the burden of melanoma is closely linked to socio-demographic development. Specifically, melanoma ranks as the 9th most common cancer in regions with a high Socio-Demographic Index (SDI) and the 2nd most common cancer (after lung cancer) in regions with a low SDI (6). While most existing studies utilizing the GBD database have focused on global macro-level reviews or analyses specific to individual countries (e.g., China or Japan), there is a notable gap in research specifically focusing on East Asia, a region of considerable global significance. Given this gap, there is a pressing need to better understand the level and trends of melanoma-related disease burden across different East Asian countries and regions. This understanding is crucial for informing evidence-based policy decisions and disease prevention strategies. The goal of this research is to provide valuable insights for policymakers in East Asia, allowing them to assess the overall disease burden in their countries and regions. This, in turn, will facilitate the development of targeted prevention strategies and ensure the equitable allocation of public health resources to address the growing challenge of melanoma in the region.

## Methods

### Data source

The global MM-related disease burden data were obtained from the GBD 2021 database at http://ghdx.healthdata.org/gbd-results-tool/ (7). Mongolia has particularities in East Asia. First, its population is less than 4 million, which is disproportionate to other countries and regions. Second, there is a significant amount of missing data about Mongolia in the GBD database. As a result, our team had to exclude Mongolia when analyzing East Asia. To systematically quantify the contribution of risk factor exposures to specific health outcomes, the Global Burden of Disease (8), Injury, and Risk Factors Study 2021 (GBD) aims to provide estimates of the full range of exposure levels, relative health risks, and attributable disease burdens for 88 risk factors in 204 countries and territories, and 811 subnational areas, from 1991 to 2021 (9). The GBD 2021 diagnosis of MM is based on the International Classification of Diseases, 10th edition (ICD-10), with codes C43-C43.9, Z85.82-Z85.828 (7).

The list of covariates used in this database for the indicators of MM Deaths, prevalence, incidence, and Disability-adjusted life-years (DALYs) can be found in the appendix of the previously published GBD 2021 paper. We used Bayesian meta-regression modeling (DisMod-MR 2.1) based on the exposure level of each risk factor disclosed in population surveys or reports to estimate the mean exposure level of that risk factor (7). A highly integrated Bayesian geospatial regression analysis tool the Cause of Death Ensemble model (CODEm) was used to predict mortality based on the available data and covariates (10).

In this study, we selected “MM” as the disease type from the database, and selected the incidence rate, prevalence rate, mortality rate, and DALYs data related to MM in East Asia (including China, Japan, South Korea, North Korea, and Taiwan Province of China) and globally from 1991 to 2021. All estimates were generated with 95% confidence intervals (95% CI), including all uncertainties due to measurement error, bias, and modeling. The upper and lower limits of the 95% CI were taken from the 97.5th and 2.5th percentiles of the 1,000 samples. The Institutional Ethics Review Board approved a waiver for this study because the data for GBD2021 are all publicly available and the study followed accurate and transparent guidelines for reporting health assessments.

### Statistical analysis

First we calculated the average annual percentage change (AAPC) and its 95% confidence interval (95% CI) using Joinpoint (National Cancer Institute, Rockville, MD, USA) (11). The core idea of the model is to build a segmented regression based on the temporal characteristics of the disease distribution, partition the study time into different intervals through several connecting points, and fit and optimize the trend for each interval, thus evaluating the characteristics of the disease change in a more detailed manner over the global time scale with different intervals of specificity (12). The Joinpoint regression model has two types of linear model (y = xb) and log-linear model (ln y = xb) (13). The data from GBD because the time of year in each country is not much, the data distribution mostly does not conform to normal distribution, and the dependent variable obeys an exponential or Poisson distribution, the log-linear model is generally chosen (10). The AAPC is calculated as {exp.(∑w_i_b_i_/∑b_i_) - 1} x 100, and we denote b_i_ as the slope coefficient for the i^th^ segment with i indexing the segments in the desired range of years, and w_i_ as the length of each segment in the range of years (14). We then used the software R (version 4.4.1) and Joinpoint software (V5.0) to count and visualize the data of this study.

The specification of this study follows TIDieR-PHP (a reporting guideline for population health and policy interventions) (15).

## Results

### Description of the burden of MM in East Asian countries and territories and worldwide

#### Incidence of MM in East Asian countries and territories and worldwide

The number of Global MM cases increased from 128,998 (95% CI: 124207–132,340) cases in 1991 to 303,105 (95% CI: 281718–318,905) cases in 2021, a percentage increase of approximately 134%. Global ASIR increased from 3.026 (95% CI: 2.907–3.108) per 100,000 population in 1991 to 3.558 (95% CI: 3.309–3.747) per 100,000 population in 2021. The AAPC for the global MM incidence rate increased by 0.5458% (95% CI: 0.3000–0.7922). The most significant increase in East Asia was in South Korea, where the number of MM cases increased from 219 (138–285) cases in 1991 to 1,553 (95% CI: 617–2055) cases in 2021, a percentage increase of approximately 576%. ASIR in South Korea increased from 0.603 (95% CI:0.389–0.789) per 100,000 population in 1991 to 1.896 (95% CI:0.78–2.499) in 2021. The AAPC of MM incidence rate in South Korea increased by 3.9143% (95% CI:3.5781–4.2516). Detailed data for the remaining countries or areas are shown in Table 1.

**Table 1:** All-age cases and age-standardized incidence, prevalence, mortality, and DALYs rates and corresponding AAPC of MM in in East Asian countries and regions and worldwide from 1991 to 2021.

Location	Measure	1991		2021		1991–2021 AAPC
		All-ages cases	Age-standardized rates per 100,000 people	All-ages cases	Age-standardized rates per 100,000 people	
		n (95% CI)				
China	Deaths	2,629 (1724–3,273)	0.305 (0.205–0.394)	5,373 (2849–7,106)	0.272 (0.145–0.358)	−0.3988(−0.6309 - -0.1663)
	DALYs	90,408 (57948–111,768)	8.926 (5.817–11.076)	153,206 (82541–204,247)	7.796 (4.209–10.369)	−0.4536(−0.6365 - -0.2704)
	Prevalence	7,648 (4841–9,447)	0.699 (0.451–0.864)	81,219 (42975–109,989)	4.157 (2.195–5.633)	6.1814 (5.7486–6.6161)
	Incidence	3,356 (2195–4,172)	0.363 (0.241–0.458)	13,437 (7198–17,979)	0.683 (0.366–0.911)	2.1448 (2.0213–2.2684)
Democratic People’s Republic of Korea	Deaths	51 (34–86)	0.304 (0.208–0.51)	107 (75–163)	0.332 (0.233–0.495)	0.2780 (0.2548–0.3012)
	DALYs	1758 (1168–2,950)	9.174 (6.163–15.284)	3,299 (2221–5,164)	9.95 (6.72–15.528)	0.2749 (0.2342–0.3157)
	Prevalence	80 (53–135)	0.407 (0.269–0.676)	163 (107–252)	0.506 (0.336–0.78)	0.7473 (0.6589–0.8357)
	Incidence	58 (39–97)	0.328 (0.223–0.552)	123 (85–186)	0.375 (0.26–0.563)	0.4471 (0.3862–0.5080)
Taiwan (Province of China)	Deaths	60 (56–63)	0.36 (0.339–0.382)	167 (151–180)	0.414 (0.374–0.445)	0.4567(−0.2111–1.1290)
	DALYs	1938 (1830–2071)	10.375 (9.781–11.049)	4,324 (3922–4,635)	11.647 (10.558–12.478)	0.3279(−0.3045–0.9643)
	Prevalence	237 (217–259)	1.164 (1.072–1.258)	796 (708–876)	2.372 (2.122–2.61)	2.3876 (1.2762–3.5111)
	Incidence	90 (84–96)	0.507 (0.475–0.542)	271 (244–292)	0.713 (0.643–0.769)	1.0561 (0.3934–1.7231)
Japan	Deaths	368 (347–379)	0.22 (0.207–0.227)	794 (669–863)	0.219 (0.198–0.232)	−0.1911(−0.5135–0.1324)
	DALYs	10,651 (10276–11,020)	6.493 (6.264–6.719)	16,767 (15100–18,061)	6.758 (6.351–7.161)	0.0132(−0.3315–0.3590)
	Prevalence	12,065 (11338–12,833)	7.462 (7.004–7.939)	33,830 (30363–36,542)	15.086 (13.993–16.055)	2.4708 (1.9132–3.0315)
	Incidence	1,603 (1504–1,698)	0.986 (0.926–1.043)	4,430 (3896–4,839)	1.798 (1.665–1.914)	1.9583 (0.9896–2.9363)
Republic of Korea	Deaths	95 (61–120)	0.306 (0.203–0.399)	235 (90–310)	0.262 (0.102–0.346)	−0.5207(−0.6814 - -0.3598)
	DALYs	3,288 (2033–4,166)	8.616 (5.524–10.864)	6,278 (2496–8,287)	7.52 (3.066–9.941)	−0.4476(−0.5766 - -0.3184)
	Prevalence	1,199 (740–1,598)	2.793 (1.767–3.764)	12,528 (4981–16,439)	15.5 (6.463–20.367)	5.8997 (5.4172–6.3843)
	Incidence	219 (138–285)	0.603 (0.389–0.789)	1,553 (617–2055)	1.896 (0.78–2.499)	3.9143 (3.5781–4.2516)
Global	Deaths	33,839 (31189–35,463)	0.849 (0.782–0.889)	61,550 (54852–66,265)	0.731 (0.652–0.787)	−0.5118(−0.6212 - -0.4023)
	DALYs	1,067,437 (980401–1,126,177)	24.302 (22.369–25.61)	1,678,836 (1474534–1,837,369)	19.632 (17.245–21.495)	−0.7320(−0.8449–0.6190)
	Prevalence	868,571 (847735–885,965)	19.531 (19.021–19.951)	2,177,566 (2057879–2,274,068)	25.37 (23.98–26.508)	0.8383 (0.6810–0.9959)
	Incidence	128,998 (124207–132,340)	3.026 (2.907–3.108)	303,105 (281718–318,905)	3.558 (3.309–3.747)	0.5458 (0.3000–0.7922)

#### Prevalence of MM in East Asian countries and territories and worldwide

Regarding prevalence, the number of Global MM cases increased from 868,571 (95%CI: 847735–885,965) in 1991 to 2,177,566 (95%CI: 2057879–2,274,068) in 2021, a percentage increase of 150%. Global ASPR grew from 19.531 (95%CI: 19.021–19.951) per 100,000 population in 1991 to 25.37 (95%CI: 23.98–26.508) per 100,000 population in 2021. The APCC of the global prevalence rate increased by 0.8383% (95% CI: 0.6810–0.9959). China had the most significant growth in East Asia, with the number of cases increasing from 7,648 (95%CI: 4841–9,447) in 1991 to 81,219 (95%CI: 42975–109,989) in 2021, a percentage increase of 961%. China’s ASPR increased from 0.699 (95%CI: 0.451–0.864) per 100,000 population in 1991 to 4.157 (95%CI: 2.195–5.633) per 100,000 population in 2021. The AAPC of the prevalence rate in China increased by 6.1814% (95% CI: 5.7486–6.6161). Detailed information for the remaining countries and regions is shown in Table 1.

#### Mortality of MM in East Asian countries and territories and worldwide

In terms of mortality, global MM deaths increased from 33,839 (95%CI: 31189–35,463) in 1991 to 61,550 (95%CI: 54852–66,265) in 2021, a percentage increase of 81%. Global ASPR decreased from 10.849 (95%CI: 0.782–0.889) per 100,000 population in 1991 to 0.731 (95%CI: 0.652–0.787) per 100,000 population in 2021. The APCC of global mortality rate decreased by 0.5118% (95%CI: −0.6212 - -0.4023). Taiwan Province of China had the most significant increase in the East Asia region, with the number of deaths increasing from 60 (95%CI: 56–63) in 1991 to 167 (95%CI: 151–180) in 2021 a percentage increase of 172%. ASMR in Taiwan province increased from 0.36 (95%CI: 0.339–0.382) per 100,000 population in 1991 to 0.414 (95%CI: 0.374–0.445) per 100,000 population in 2021. APCC for mortality in Taiwan province increased by 0.4567 (95%CI: −0.2111–1.1290). Detailed information for the remaining countries and regions is shown in Table 1.

#### DALYs of MM in East Asian countries and territories and worldwide

The DALYs for MM globally grew from 1,067,437 (95%CI: 980401–1,126,177) in 1991 to 1,678,836 (95%CI: 1474534–1,837,369) in 2021, a percentage increase of 57%. Global ASDR decreased from 24.302 (95%CI: 22.369–25.61) per 100,000 population in 1991 to 19.632 (95%CI: 17.245–21.495) per 100,000 population in 2021. Global APCC for DALYs decreased by 0.732% (95%CI: −0.8449 - 0.6190). Taiwan Province of China had the most significant growth in East Asia with the DALYs increasing from 1938 (95%CI: 1830–2071) in 1991 to 4,324 (95%CI: 3922–4,635) in 2021 a percentage increase of 123%. ASDR in Taiwan province grew from 10.375 (95%CI: 9.781–11.049) per 100,000 population in 1991 to 11.647 (95%CI: 10.558–12.478) per 100,000 population in 2021. The APCC for DALYs in Taiwan province increased by 0.3279 (95%CI: −0.3045 - 0.9643). Detailed information for the remaining countries and regions is shown in Table 1.

### Joinpoint regression analysis of the burden of MM in East Asian countries and territories and worldwide

**Figure 1:**
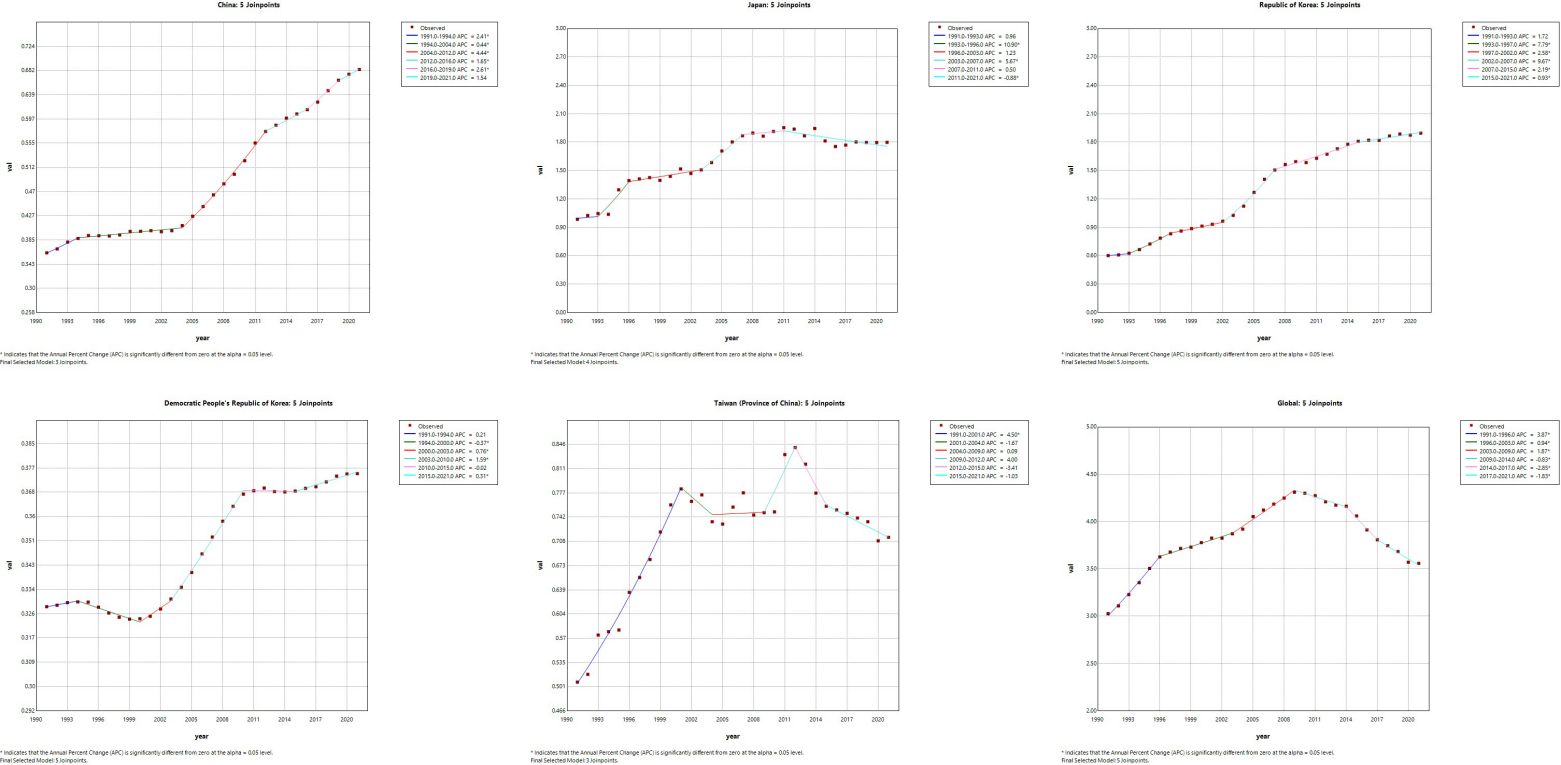
The APC of ASIR of MM in East Asian countries and regions and worldwide from 1991 to 2021 (*means *p*-values < 0.05 and significant results).

**Figure 2:**
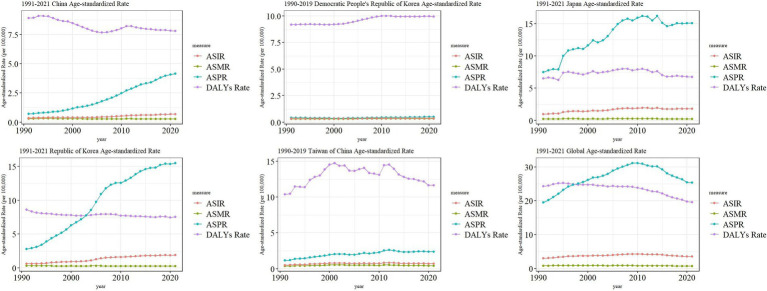
The APC of ASPR of MM in East Asian countries and regions and worldwide from 1991 to 2021 (*means *p*-values < 0.05 and significant results).

**Figure 3:**
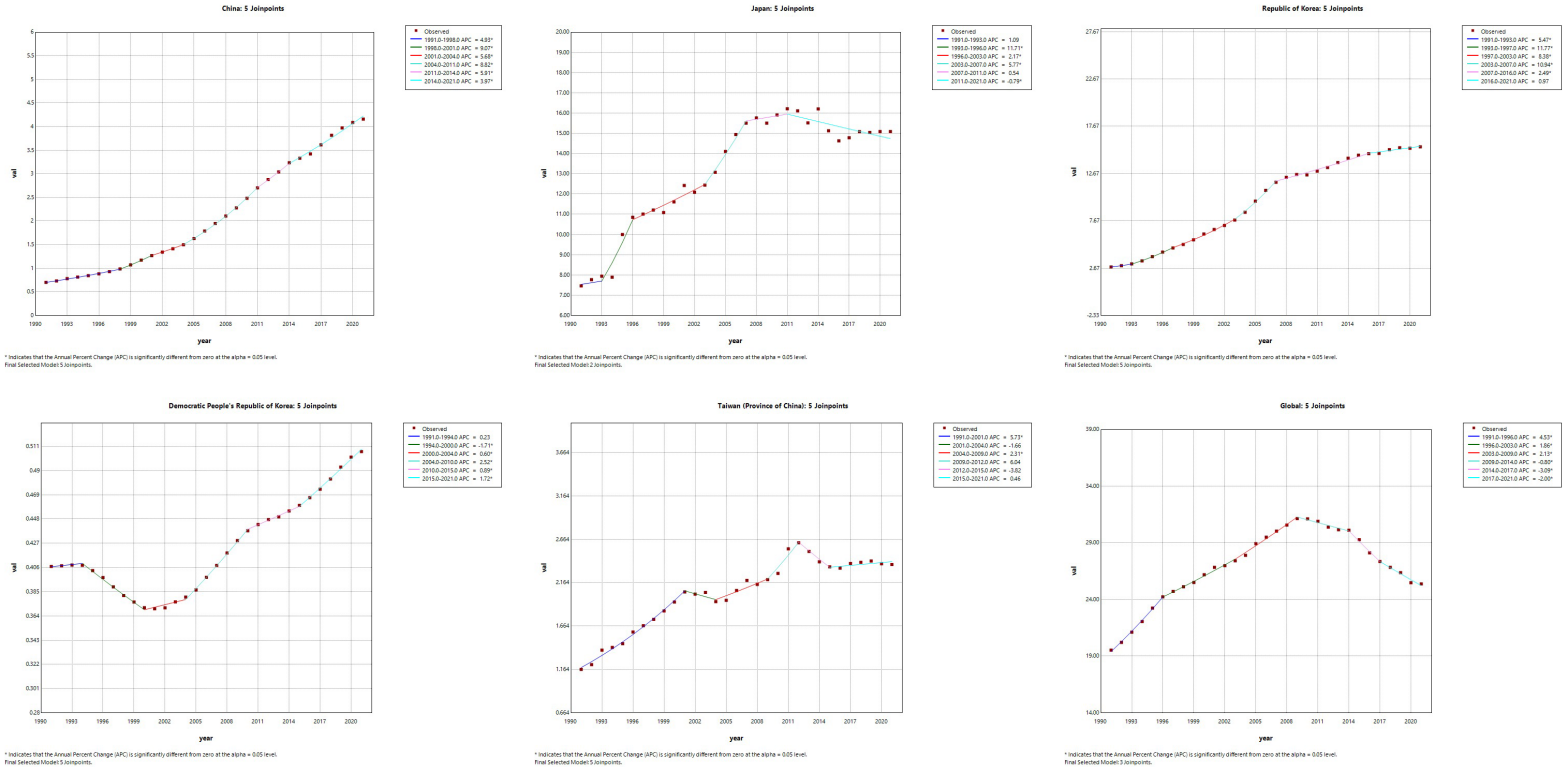
The APC of ASMR of MM in East Asian countries and regions and worldwide from 1991 to 2021 (*means *p*-values < 0.05 and significant results).

**Figure 4:**
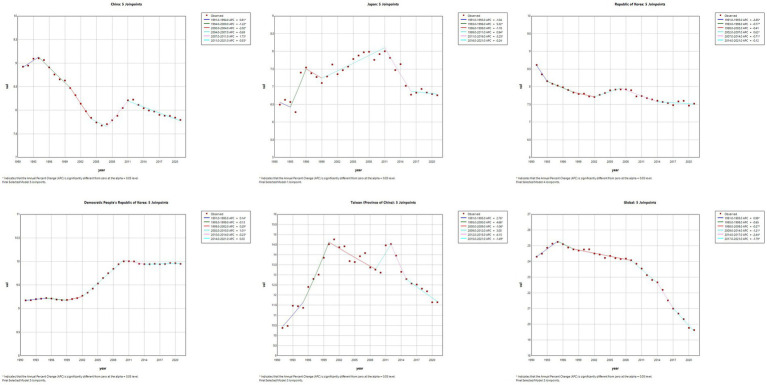
The APC of ASDR of MM in East Asian countries and regions and worldwide from 1991 to 2021 (*means *p*-values < 0.05 and significant results).

The results of the Joinpoint regression analysis of ASIR, ASPR, ASMR, and ASDR for East Asian countries and regions from 1991 to 2021 can be seen in Figures 1–4. Let us take China as an example, the annual rate of change (APC) of ASIR is in an increasing trend from 1991–2021, and there is a rapidly increasing trend after 2004 that lasts until 2012 (1991–1994 APC = 2.41; 1994–2004 APC = 0.44; 2004–2012 APC = 4.44; 2012–2016 APC = 1.65; 2016–2019 APC = 2.61; 2019–2021 APC = 1.54; *p* < 0.05). The APC of China’s ASPR had a steady growth trend from 1991–2021 had a steady growth trend from 1991–2021 (1991–1998 APC = 493; 1998–2001 APC = 9.07; 2001–2004 APC = 5.68; 2004–2011 APC = 8.82; 2011–2014 APC = 5.91; 2014–2021 APC = 3.97; *p* < 0.05). However, the global APC of ASIR and ASPR had an increasing trend from 1991–2009 and a decreasing trend from 2009–2021. The APC of ASMR and ASDR in China had an increasing trend in 1991–1994 and 2006–2011, and a decreasing trend in 1994–2006 and 2011–2021, but the APC of ASMR and ASDR globally had an increasing trend in 1991–1995 and a decreasing trend in 1995–2021.

### Trends in the burden of MM in East Asian countries and territories and worldwide

**Figure 5:**
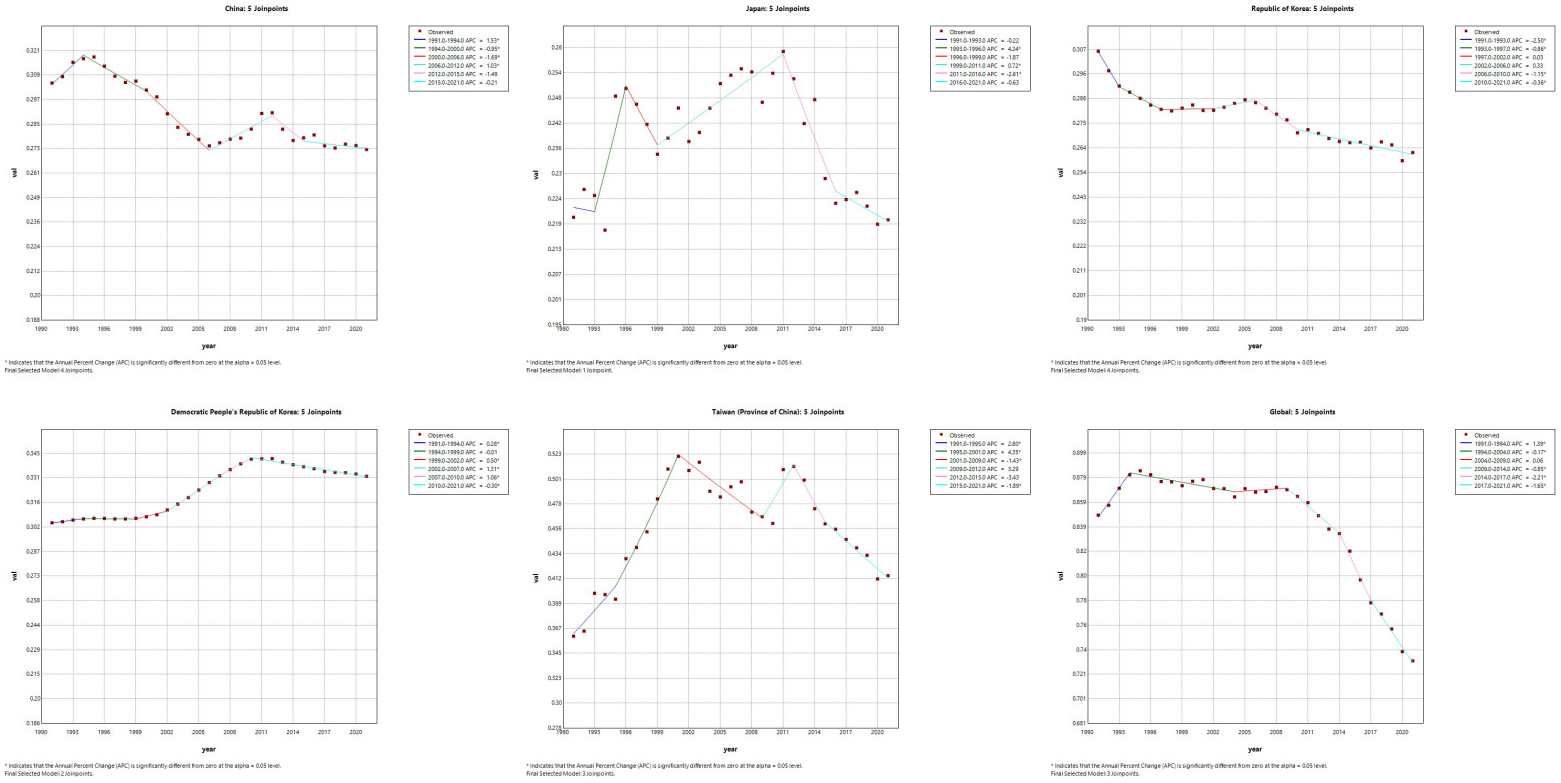
Trend comparison of ASIR, ASPR, ASMR, and ASDR of MM in East Asian countries and regions and worldwide from 1991 to 2021.

We found that the ASIR of MM in East Asia all showed a slowly increasing trend, and the global MM ASIR showed an increasing trend from 1991–2010 and a decreasing trend from 2010–2021. The ASPR of MM in China, North Korea, South Korea, and Taiwan showed a gradual upward trend in 1991–2021, while the ASPR of global MM was elevated in 1991–2010 and decreased in 2010–2021. However, the ASPR of Japanese MM reaches a small peak in 2001, 2008, 2011, and 2014, respectively, with a higher overall fluctuation. The ASMR of both East Asian and global MM did not show large changes. The ASDR of China, South Korea, and Global MM showed an overall decreasing trend from 1991–2021, North Korea was a slightly increasing trend, while Japan and Taiwan province were more fluctuating (Figure 5).

### The burden of MM in different age groups in East Asian countries and territories in 1991 and 2021

**Figure 6:**
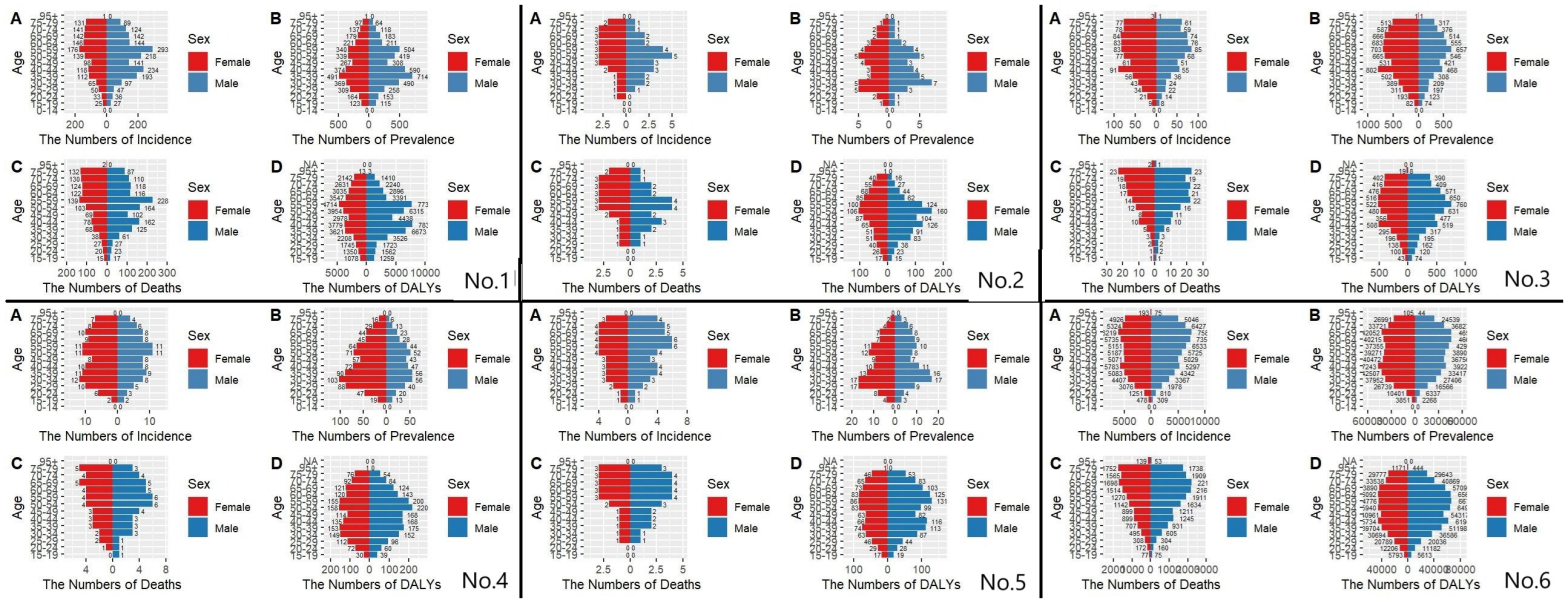
Comparative of the incidence, prevalence, deaths, and DALYs counts, along with their crude rates, by age group in East Asian countries and regions and worldwide from 1991 to 2021. **(A)** Incident cases and CIR; **(B)** Prevalent cases and CPR; **(C)** Death cases and CMR; **(D)** DALYs counts and CDR; Bar charts represent counts; lines represent crude rates; (No.1) China; (No.2) North Korea; (No.3) Japan; (No.4) South Korea; (No.5) Taiwan of China; (No.6) Golbal.

The results of the present study (Figure 6) showed that, from the results of the incidence rate, the number of people with incidence peaked at 50–70 years of age in 1991 in East Asia and globally, and the same number of people with incidence in 2021 in East Asia (except Japan), while the number of people with incidence in 2021 in Japan and globally peaked at 70–74 years of age. CIR in East Asia and globally generally showed an increasing trend with age in 1999 and 2021. Regarding the prevalence in 1991, we found that East Asia and the world reached the first peak at 30–34 years of age and the second peak at 50–60 years of age. The peaks of the prevalence in 2021 varied considerably in China and Taiwan Province of China (55–59 years of age), North Korea (50–54 years of age), Japan and the world (70–74 years of age), and South Korea (60–64 years of age). The trend of 1991 and 2021, the trend of CPR in East Asia (except North Korea) and globally showed an increase and then a decrease, while the trend in North Korea was generally increasing. For 1991 mortality, the peak age groups were (55–59 years) in China, (55–59 years) in North Korea, (75–79 years) in Japan, (55–59 years) in South Korea, (60–64 years) in Taiwan Province of China, and (65–69 years) globally. 2021 mortality rates were (55–59 years) in China, (55–59 years) in North Korea, (85–54 years) in Japan, and (85–54 years) in South Korea (75–79 years), Taiwan Province of China (65–69 years), and globally (70–74 years). The overall profile of DALYs is similar to that of mortality.

### Gender disparities in the burden of MM in different age groups in East Asia

**Figure 7:**
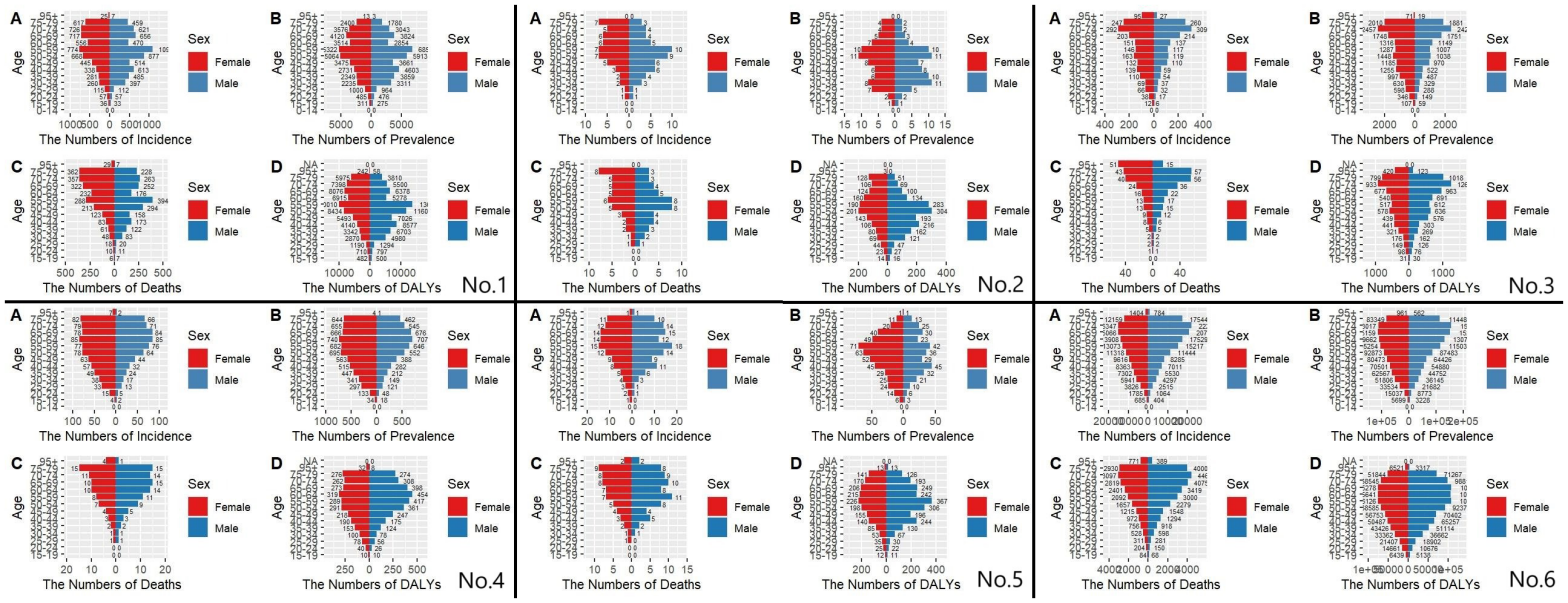
Comparison of the number of incidence, prevalence, mortality, and DALYs of MM in males and females of different age groups in East Asian countries and regions and worldwide in 1991. **(A)** Incidence; **(B)** Prevalence; **(C)** Mortality; **(D)** DALYs; (No.1) China; (No.2) North Korea; (No.3) Japan; (No.4) South Korea; (No.5) Taiwan of China; (No.6) Golbal.

**Figure 8:**
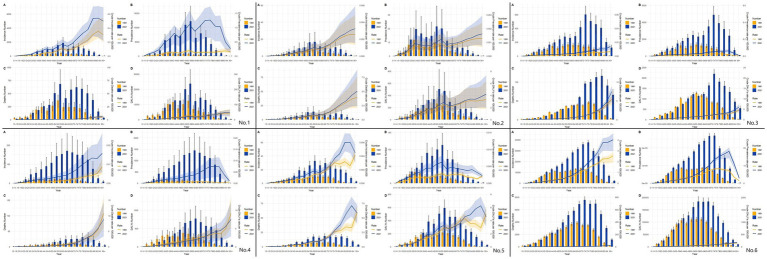
Comparison of the number of incidence, prevalence, mortality, and DALYs of MM in males and females of different age groups in East Asian countries and regions and worldwide in 2021. **(A)** Incidence; **(B)** Prevalence; **(C)** Mortality; **(D)** DALYs; (No.1) China; (No.2) North Korea; (No.3) Japan; (No.4) South Korea; (No.5) Taiwan of China; (No.6) Golbal.

Figures 7, 8 show the incidence-prevalence-mortality and DALYs for males and females in different age groups in East Asia and globally in 1991 and 2021, respectively. For the incidence in China in 1991 and 2021, we found that the number of incidence was highest in the age group of 55–59 years, and the number of incidence was significantly higher in males than in females before the age of 60 years, and the number of incidence in males was lower than that in females after the age of 60 years, which is also consistent with previous studies. 1991 global prevalence is highest in the 65–69 age group, with significantly fewer males than females before age 45 and more males than females after age 45. 2021 global prevalence is highest in the 70–74 age group, with significantly fewer males than females before age 50 and more males than females after age 50. For the 1991 prevalence rate, we take Japan as an example, and we find that except for a peak at 40–44 years of age, all the other age groups have an increasing trend until 60 years of age, and a decreasing trend after 60 years of age, with more females than males. The prevalence rate in Japan in 2021 is similar to that of 1991, but it peaks at 70–74 years of age. As for the DALYs, the DPRK in 1991 and 2021 is peaking at age 50–54, with an increasing trend until age 55 and a decreasing trend after age 55. However, in 1991, there were more males than females between the ages of 30–60 and vice versa in the rest of the age groups, while in 2021, there were more males than females before the age of 60 and more females than males after the age of 60.

**Figure 9:**
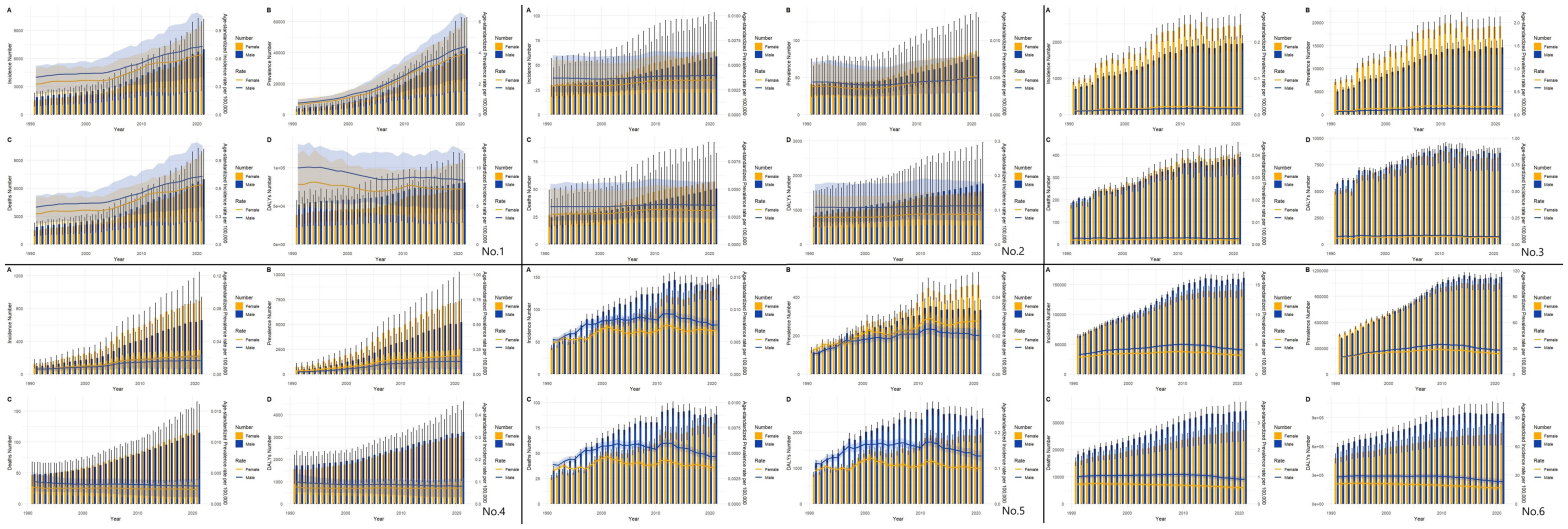
Comparison of full-age cases and age-standardized rates of incidence, prevalence, mortality and DALYs among men and women in East Asian countries and regions and worldwide. **(A)** Incident cases and ASIR; **(B)** Prevalent cases and ASPR; **(C)** Death cases and ASMR; **(D)** DALYs counts and ASDR. Bar charts represent counts; lines represent age-standardized rates; (No.1) China; (No.2) North Korea; (No.3) Japan; (No.4) South Korea; (No.5) Taiwan of China; (No.6) Golbal.

Figure 9 illustrates the burden of disease versus age-standardized incidence of MM among men and women of all ages in East Asia and globally from 1991–2021. We found that the overall trend of ASIR varies considerably among countries and regions, with an increasing trend in South Korea, and China, a relatively stable trend in North Korea and Japan, and an increasing and then decreasing trend in Taiwan province, and globally, but with greater fluctuations in South Korea. However, there are more female ASIR than male ASIR in Japan and South Korea, and more male ASIR than female ASIR in the rest of the countries and regions. The overall trend of ASPR in East Asia and globally from 1991–2021 is also similar to that of ASIR, with more females than males in Japan, Korea, and Taiwan province (post-1996), and more males than females in Korea, Taiwan province, and globally. Regarding ASDR and ASMR for the years 1991–2021, the trends in China are diametrically opposite ASMR is increasing and ASDR is decreasing. North Korea and Japan are generally more stable, while South Korea and the globe both show a slight downward trend. Both China and Taiwan are also still up and then down, with more volatility. We find that there are large differences between men and women in China, Taiwan, and Global and that men have more ASDR and ASMR than women.

## Discussion

This study provides the first comprehensive assessment of the temporal trends in malignant melanoma (MM) burden across East Asian countries and regions (China, Japan, South Korea, North Korea, and Taiwan Province of China) from 1991 to 2021, revealing striking disparities in incidence, prevalence, mortality, and disability-adjusted life years (DALYs). Key findings include: (1) South Korea exhibited the most rapid rise in MM incidence (AAPC: 3.91%, 1991–2021), while China experienced the steepest increase in prevalence (AAPC: 6.18%), reflecting divergent epidemiological trajectories driven by socioeconomic and environmental factors; (2) Taiwan Province of China demonstrated the highest escalation in mortality and DALYs (ASMR: +15.0%, ASDR: +12.3%), underscoring unmet clinical needs in early detection and treatment; (3) Age-specific burden peaks varied markedly—earlier mortality peaks in lower-SDI regions (e.g., China at 55–59 years) versus delayed peaks in high-SDI areas (e.g., Japan at 85–89 years), highlighting the interplay of aging, healthcare access, and prevention prioritization. These findings fill a critical gap in regional MM burden analysis and emphasize the necessity of tailored public health strategies to address heterogeneities in East Asia.

Our analysis showed that from 1991 to 2021, the ASIR, ASPR, and ASMR of MM patients in China were on an upward trend, but their ASDR was on a downward trend. The fluctuation of these four indicators in North Korea and Japan was not significant. In South Korea, ASIR and ASPR are rising and ASMR and ASDR are decreasing. Taiwan province and the world showed a rising and then falling trend, but Taiwan province had greater fluctuations. The prevalence morbidity and mortality rate of men in China, Taiwan, and the world is greater than that of women, whereas in South Korea, North Korea, and Japan there are more women than men. This suggests that there are large differences in the burden of disease for MM among East Asian regions. Among different age groups, we found that ASIR, ASPR, ASMR, and ASDR all peaked in the middle-aged and older age groups, but there were significant differences in different countries or regions, and in terms of the 1991 mortality rate, the peak age groups were (55–59 years) in China, North Korea and South Korea, (75–79 years) in Japan, (60–64 years) in Taiwan Province of China, (65–69 years) globally. In 2021, the peak age groups were North Korea, China (55–59 years), Japan (85–89 years), South Korea (75–79 years), Taiwan Province of China (65–69 years), and globally (70–74 years). Regarding the differences in mortality rates between regions, the great influence of sociodemographic indices on MM patients can be maximized. Trying to compare countries or regions with low to medium SDI like China and North Korea, countries or regions with high SDI like South Korea, Taiwan province, and especially Japan tend to have their peak age group of MM mortality occurring at 60–70 years old or even later, while China and North Korea MM mortality occurs after 50 years old (16). Of course, the effect of social aging cannot be excluded, but with the progress of social aging in China, this reason can no longer explain this phenomenon. This phenomenon can be explained by the fact that melanoma prevention and control are less likely to be a high priority due to economic constraints, especially in poorer areas, leading to this discrepancy (17).

This shows that the peak age of death of MM patients in developed regions such as Japan and Korea has been well delayed under the current situation. Therefore, we not only need to revisit the previous health strategies for MM but also propose new effective strategies to control the burden of disability of MM in response to regional differences.

Epidermal keratinocytes take up melanin and use it to protect their nuclei from UV radiation-induced DNA damage (18). Melanoma is a malignant tumor caused by the transformation and uncontrolled proliferation of melanocytes. Melanocytes from different sites can malign into different phenotypes of melanoma, of which cutaneous melanoma can account for more than 90% (19). Over the past many years, most studies on the global disease burden of malignant cutaneous melanoma have focused on developed regions such as Europe, the United States, Australia, and New Zealand (13). However, due to ethnic differences, the high-risk phenotype of European origin (fair skin, hair, and eye color), combined with a tanning culture that favors high exposure to UV rays during sunny holidays and indoor tanning (20), has led to a marked differentiation in the MM disease burden between populations in these regions and those in East Asia, where the incidence rate is much lower but the mortality rate of MM in East Asia is high and disproportionately, which is worth exploring and analyzing (21). In recent years, a large number of Chinese researchers have also analyzed the burden of disease of MM in China, and their studies are also of great reference significance to us, but no studies are focusing on comparisons between various countries and regions in East Asia, and the present study fills the gap in this region very well.

Our study showed that the highest increase in incidence in East Asia from 1991 to 2021 was in South Korea, the highest increase in prevalence was in China, and the highest change in mortality and DALYs was in Taiwan province. As to why such differences occur, we should make a comprehensive consideration that includes multiple factors. Compared to South Korea and Taiwan, there was a rapid economic boom from the 1960s to the 1990s, and their societies as a whole had already reached a certain level of development by the end of the last century (in the case of Japan, the period of rapid development was much earlier) (22). China, on the other hand, only began its rapid economic progress in the 1990s, and by the 2020s, there was still a gap between its overall social level and that of developed regions such as Japan and South Korea. North Korea, on the other hand, remains relatively underdeveloped to this day. Numerous studies have demonstrated that the higher burden observed in older adults in areas of high sociodemographic indices (SDI) may be due in part to a peak in morbidity resulting from structural and physiologic decline due to aging (23). Studies have shown that more than 80% of melanomas can be attributed to UV exposure in South Korea (24), North Korea, and Japan, which as a whole are located in higher latitudes, Taiwan in the subtropics, and China, whose latitude even encompasses the temperate, subtropical and tropical zones due to its large size. Therefore, the higher incidence of MM patients in Taiwan province is also related to its longer and more intense direct sunlight (25). MM is innately sensitive to UV light, and the great geographic variability of MM is tied to latitudinal differences. Closer to the equator means more UV radiation (26). The risk of MM increases by 10 percent for every 2-meter rise in elevation, which is attributed to the fact that at higher elevations, the air through which the sunlight passes is thinner, increasing the level of UV exposure in the population. Urbanization and higher (27). Ultraviolet (UV) radiation remains a critical modifiable risk factor. High-latitude regions like Japan and South Korea experience seasonal UV peaks, whereas Taiwan’s subtropical climate entails year-round exposure, correlating with its elevated age-standardized mortality rate. Rapid urbanization in economies like China has led to increased outdoor occupational exposure and lifestyle changes, further amplifying risk.

The effect of gender on melanocytes has been verified in many previous studies, with a significantly lower global prevalence in men than in women up to the age of 50, and a higher global prevalence in men than in women after the age of 50 (28). Overall, more men than women develop melanoma, which may be related to different behavioral patterns between men and women, e.g., women tend to be more aware of the use of sunscreen (29). Although the prevalence of MM is higher in women before the age of 50, their prognosis tends to be better, which is related to the fact that estrogen can block the inhibitory pathway that prevents the recognition of melanoma, thus stimulating the elicitation of an immune response (30). Meanwhile, the prevalence of MM before the age of 30 is maintained at a high level in all countries and regions, and although the peak of the disease is still concentrated in the middle-aged and old age, we can still judge that MM is the most common malignant tumor in the adolescent group (31).

Genetic factors play a huge role in the disease burden of malignant melanoma in East Asia. East Asian populations exhibit distinct genetic profiles influencing MM susceptibility. For instance, mutations in the CDKN2A gene, commonly observed in Caucasian melanoma patients, are rare in East Asia. Instead, mutations in the KIT gene and the predominance of acral lentiginous melanoma subtypes are more common, particularly in China and Japan. These genetic differences may explain the lower overall incidence but higher mortality rates in East Asia, as acral melanomas are often diagnosed at advanced stages.

An interesting finding in our study is that both prevalence and incidence reach their peak after 50 years of age in most of the East Asian region, but before that, a sharp rise, the first mini-peak, is ushered in at the age of 30–40 years (32). Concerning this phenomenon, we hypothesize that the cause may be related to the interaction of a higher frequency of hazardous exposures with a gradual decline in physical status. But its exact cause, which so far has not been studied by any team, needs to be further explored.

Lifestyle and socioeconomic factors also have a huge impact on the disease burden of malignant melanoma in East Asia. 1. Sun Protection Measures: Gender disparities in MM burden, such as higher male mortality in China, may reflect behavioral differences. Studies indicate that East Asian women adopt sun-protective behaviors (e.g., sunscreen use, parasol use) more consistently than men. 2. Healthcare Accessibility: Regions with higher Socio-demographic Index (SDI), such as Japan and South Korea, benefit from early detection programs and advanced therapies, delaying mortality peaks to older ages. In contrast, limited access to dermatologic care in rural China or North Korea delays diagnoses, resulting in earlier mortality peaks. 3. Aging Populations: Japan’s super-aged society, with mortality peaks at 85–89 years, underscores the interplay between demographic shifts and MM burden, whereas younger populations in lower-SDI regions face competing health priorities. 4. Cultural Practices: The popularity of skin-whitening products in East Asia may reduce UV exposure but also delay lesion detection due to altered pigmentation. These multifactorial influences—genetic predisposition, UV exposure gradients, healthcare inequities, and cultural behaviors—collectively shape MM trends in East Asia. Our findings align with global studies but highlight region-specific nuances, such as the predominance of non-UV-driven subtypes. Future research should explore gene–environment interactions and evaluate targeted prevention strategies, such as public awareness campaigns for acral melanoma in high-risk populations.

The GBD database provides high-quality estimates of the global cancer burden, but some shortcomings remain (33). Limitations of this study: Because GBD 2021 only covers the burden of disease indicators at the national and regional levels (34), it is not possible to further analyze the MM-related data of China’s provinces to clarify the differences in the burden of MM among China’s regions. Secondly, the GBD database has deficiencies in data quality and timeliness, and there are inevitable discrepancies in the fitting of unavailable data. The accuracy and completeness of the GBD data are contingent upon the quality and availability of primary sources from each country or region. In certain low-and middle-income areas, including parts of East Asia, data may be incomplete or missing, leading to potential estimation biases. For instance, Mongolia was excluded from our analysis due to significant data gaps and population size disparities. Similarly, subnational areas within China or North Korea lack granular data, which could result in underestimation or overestimation of disease burden in these regions, especially in under-resourced settings with limited surveillance infrastructure. However, the GBD employs advanced modeling tools, such as DisMod-MR 2.1 and CODEm, to address data gaps. Despite these efforts, some degree of uncertainty remains, which may influence the precision of our findings. Therefore, interpretations, particularly for regions with sparse data, should be approached with appropriate caution. Differences in diagnostic methods between countries or regions may also affect our analysis. For example, Japan and South Korea have advanced healthcare systems with standardized melanoma diagnostic protocols (e.g., dermoscopy, biopsy practices), whereas rural areas in China or North Korea may rely on less precise clinical assessments. These discrepancies could inflate reported incidence rates in high-resource regions while undercounting cases in low-resource settings. These limitations may affect cross-country comparability and trend accuracy. However, the GBD’s standardized methodology helps mitigate some biases by harmonizing data inputs. Our findings align with prior studies, suggesting that the overall trends remain valid despite regional data limitations. Future studies should prioritize subnational data collection to enhance granularity and accuracy.

This study has several strengths: First, it is the first comprehensive assessment of the burden of disease associated with MM in East Asia today and globally, and is based on the most comprehensive review of available data. This study provides an evidence-based assessment of the effectiveness of current prevention strategies and informs the development of future prevention and control policies (35). In addition, there may also be differences in the number of deaths and ASMR associated with different parts of the body, such as limb MM versus facial MM, yet there are currently no data related to deaths for these two subtypes in the GBD database. Therefore, in the future, the trend of disease burden within different countries should be further evaluated and further studies and investigations for MM should be conducted to supplement the acquisition of better data and information. This will help guide the development of specific prevention and control policies in different countries and regions to reduce the burden of melanoma.

## Conclusion

From 1991 to 2021, significant regional differences in the burden of malignant melanoma (MM) were observed in East Asia. Countries with higher socio-demographic indices (SDI), like South Korea, Japan, and Taiwan, showed delayed peaks in MM mortality, while regions with lower SDI, such as China and North Korea, experienced earlier mortality peaks. The findings highlight the impact of socio-economic factors, UV exposure, and healthcare access on MM trends. These disparities underscore the need for tailored prevention and control strategies to reduce the melanoma burden in different East Asian regions.

## Data Availability

The original contributions presented in the study are included in the article/supplementary material, further inquiries can be directed to the corresponding author.
